# The effects of heart rate variability biofeedback on functional gastrointestinal disorders: a scoping review

**DOI:** 10.3389/fphys.2025.1511391

**Published:** 2025-05-22

**Authors:** Ashley G. Pereira, Lily Fu, William Xu, Armen A. Gharibans, Greg O’Grady

**Affiliations:** ^1^ Department of Surgery, The University of Auckland, Auckland, New Zealand; ^2^ Alimetry Ltd, Auckland, New Zealand; ^3^ Auckland Bioengineering Institute, The University of Auckland, Auckland, New Zealand

**Keywords:** heart rate variability, functional gastrointestinal disorder, biofeedback, disorder of gut brain interaction, resonance breathing

## Abstract

**Background:**

Functional Gastrointestinal Disorders (FGID) are a group of symptom-based disorders that occur across the alimentary tract and have a high prevalence globally in both adults and children. These symptoms are chronic and/or recurrent and often have substantial effects on quality of life. Their incidence is tied to multiple factors, including gut-brain axis imbalance, which includes autonomic dysregulation related to a relative withdrawal of vagal activity. Heart rate variability biofeedback (HRVB) is a non-invasive intervention that can influence autonomic activity and has shown benefit for diverse conditions including depression and anxiety, however the evidence of its effect has not yet been systematically assessed in FGIDs. This scoping review aimed to collate and evaluate the available literature regarding HRVB and FGIDs.

**Methods:**

We systematically searched four medical databases. Four interventional studies using HRVB in FGIDs met inclusion criteria.

**Results:**

Studies were heterogeneous, including both paediatric and adult patients, as well different subtypes of FGID. Two of the four studies demonstrated significant symptom improvements from HRVB while the other two found no significant difference.

**Discussion:**

Our findings suggested that at least 6 weeks of HRVB is required to observe an impact on FGID symptoms. We provide recommendations for future studies of HRVB in FGIDs, which are needed. Evidence on HRVB for FGID is still emerging, but appears promising when administered optimally.

## 1 Introduction

Functional Gastrointestinal Disorders (FGID), more recently termed Disorders of Gut-Brain Interactions (DGBIs), are a group of multiple symptom phenotypes that occur across the gastrointestinal (GI) tract. There are several subtypes and symptoms range from dysphagia to dyspepsia to abdominal pain and bloating (Black et al., 2020; Sperber et al., 2021). These disorders may have recurrent and potentially debilitating impacts, and an incomplete understanding of their pathophysiology means that clinical diagnosis and treatment often still rely upon trial and error. They are highly prevalent, affecting up to 40% of the global population (Black et al., 2020; Sperber et al., 2021; Andreasson et al., 2021).

FGIDs can be better understood through the Biopsychosocial model of disease (Black et al., 2020; Van Oudenhove et al., 2016). This model emphasises that the development and persistence of FGIDs are shaped by an interplay of physiological, psychological, and environmental factors. Recognising this complexity is essential for effective management, as it highlights the need for a holistic, multifactorial approach that goes beyond physiological symptoms to consider social and psychological influences as well.

Recently, there has been increasing evidence of a correlation between the prevalence of functional gut symptoms and an imbalance of autonomic nervous system activity, with greater relative sympathetic activity due to parasympathetic withdrawal or a decrease in cardiovagal modulatory ability (Aggarwal et al., 1994; Bharucha et al., 1993; Chelimsky et al., 2019; Mróz et al., 2022; Jung-Ho et al., 1999). This hypothesis is supported by the emerging efficacy of therapies proposed to enhance vagal tone, encompassing such diverse approaches as chewing gum, slow breathing exercises, moderate-pressure massage, or transcutaneous vagal electrical stimulation (Lunding et al., 2008; Zhang et al., 2023), accompanied by with evidence for improved antral, colonic and oesophageal motility and symptom reductions (Bonaz et al., 2016). This decrease in vagal modularity represents one possible physiological cause for FGID as per the biopsychosocial model.

**FIGURE 1:**
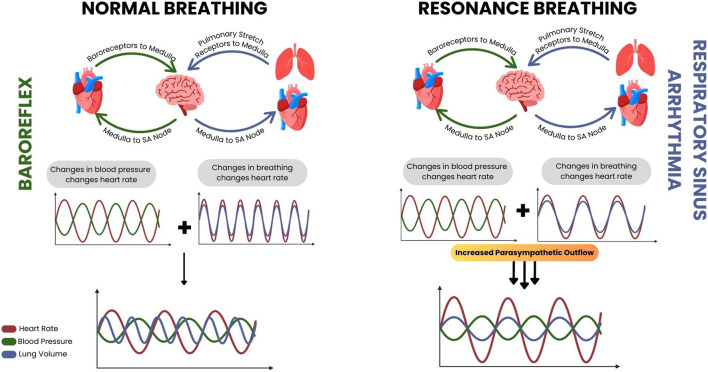
A diagrammatic resource developed by Pereira et al. to demonstrate the physiology underlying heart rate variability biofeedback. During normal breathing conditions (left) the resultant heart rate changes from the baroreflex and respiratory sinus arrhythmia are out of sync, but while conducting resonance breathing during biofeedback (right) the resultant heart rate changes from the respiratory sinus arrhythmia approaches that of the baroreflex and they resonate, along with the parasympathetic activity that occurs alongside them.

Heart Rate Variability Biofeedback (HRVB) is a non-invasive technique that leverages the body’s physiology and autonomic regulation, using specific slow breathing rates to modulate heart rate and enhance baroreflex sensitivity (Lehrer and Gevirtz, 2014). HRVB is often stated to be carried out at one’s resonance frequency, the frequency of breathing where the oscillation of heart rate due to the respiratory sinus arrhythmia (produced by the slow breathing) resonates with the oscillation in heart rate due to the baroreflex (Figure 1). This results in a maximal heart rate variation, and a maximal increase in baroreflex gain with an increase in baroreflex gain at resting states after consistent practice as well (Lehrer et al., 2003; Shaffer, 2020; Vaschillo et al., 2006; Wheat and Larkin, 2010).

This ability to influence the activity of the autonomic nervous system could have therapeutic significance for FGIDs, but existing evidence on the effect of HRVB on the GI tract is limited. In addition, standardised protocols for performing HRVB in FGIDs have not been defined Although the most common protocol is that of Lehrer et al. (Lehrer et al., 2013), there still exists considerable variation.

The aim of this study was therefore to examine the role and effectiveness of HRVB as a therapy for individuals with FGIDs through a scoping review. The primary aim was to identify and assess relevant interventional clinical studies applying HRVB in populations with FGID and assessing their relevant symptoms. The secondary aims were to assess the protocols and measurement tools used by each study, while comparing the study’s outcomes, in order to develop a protocol for future studies to measure the effect of biofeedback on patients diagnosed with FGIDs, and to guide future research in this emerging area. As this is a scoping review in an emerging field, we aim to map the existing literature and identify gaps to guide further studies.

## 2 Methods

### 2.1 Study design

The scoping review was conducted and reported in accordance with the PRISMA 2020 guidelines and the scoping review extension (PRISMA-ScR), and thus, did not adhere strictly to PICOS guidelines (Page et al., 2021; Tricco et al., 2018).

### 2.2 Search strategy and study selection

Four databases were searched: PubMed, Web of Science, ScienceDirect and Scopus; using the search term ‘Heart Rate Variability’ alongside terms to describe categories and terminologies for FGIDs as defined by ROME IV (Rome IV Criteria, 2020), including “Functional abdominal pain”, “Nausea and Vomiting Syndromes”, “Functional Dyspepsia”, “Gastroparesis”, “Irritable Bowel Syndrome”, “Functional Constipation”, “Functional Diarrhoea”, and “Functional abdominal bloating” (i.e., “Heart Rate Variability AND Functional Dyspepsia). The literature search was completed on 8 January 2024.

It was decided to use the search term ‘heart rate variability’ as opposed to ‘heart rate variability biofeedback’ as it was a broader search term, and many studies did not use this term in their work, instead opting for ‘slow deep breathing’ or similar phrases to describe a similar technique where heart rate variability (HRV) is measured and altered due to a breathing technique intervention. This consistent measurement of HRV across studies included, allowed for greater rigor when comparing interventions and their effect on autonomic activity. Individual searches were completed for each category of FGID, as opposed to simply searching FGID, so as to ensure adequate collection of studies in the search.

Two reviewers, who were not associated with Alimetry Ltd, independently screened the literature titles, abstracts and then the entire article according to the inclusion and exclusion criteria detailed in Table 1. Reviewers specifically confirmed that HRV was measured as part of the study so as to allow for a consistent comparison across literature, although there were no limits on the method by which HRV or gastrointestinal symptoms were measured. Studies that used electrocardiogram (ECG) and photoplethysmography (PPG). were both collected as both were proved to be equivalent to each other as justified by Plews et al. (2017), as well as studies that used both symptom questionnaires and concurrent clinical investigations. Literature that was published in languages other than English were excluded from this study due to limited translation resources and language proficiency within the research team. This ensured that accurate data extraction and synthesis was conducted on all included literature.

**TABLE 1:** The key inclusion and exclusion criteria that was used to screen all relevant articles.

Inclusion criteria	Exclusion criteria
Measures heart rate variability	Does not measure Heart Rate Variability
Intervention of HRVB or similar as per Lehrer et al., 2013	Gastrointestinal syndromes of known physiological cause (non FGIDs)
Published within the last 10 years (2014 onwards)	Intervention is not solely heart rate variability biofeedback or auricular transcutaneous electrical stimulation
	Articles whose clinical focus is outside the scope of the search
	Observational Studies
	Conference abstracts and review articles
	Articles in languages other than English

FGID (Functional Gastrointestinal Disorders).

HRVB (Heart Rate Variability Biofeedback).

### 2.3 HRV metrics and biomarkers

**FIGURE 2:**
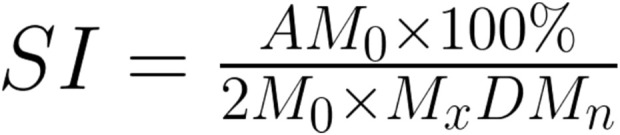
Baevsky Stress Index Calculation. M_0_ is the mode, AM_0_ is the mode amplitude calculated using a 50 m bin width, M_x_DM_n_ is the difference between the longest (M_x_) and the shortest (M_n_) interval (Baevsky and Chernikova, 2017).

There are several different metrics that can be used to assess HRV, and the following considerations were incorporated into the review. HRV metrics are primarily divided into Time Domain and Frequency Domain Measures (Shaffer and Ginsberg, 2017). The Time Domains largely focus on the interbeat intervals (IBI) defined as the time between each successive heartbeat, displaying the variance in these successive intervals. The two most commonly used metrics for this are SDNN (Standard Deviation of the N to N Intervals or normal R-R intervals without artefact) and RMSSD (Root Mean Square of the Successive Differences). There are other variables included within the time domain measurements which were not relevant to the scope of this review. The Frequency Domain Measures rely on the ability to conduct a Fast Fourier Transform (FFT) on the heart rate data, separating the data into three separate bands: high frequency, low frequency, and very low frequency (HF, LF and VLF, respectively). Each of these frequency bands cover a set range of frequencies: 0.15–0.40 Hz for HF, 0.04–0.15 Hz for the LF, and 0.0033–0.04 Hz for VLF, and they are expressed as a power within those frequency bands. Of these frequency metrics, LF originally is thought to represent the sympathetic arm of the baroreflex, and through its arterial oscillations (known as Mayer’s waves), stabilises blood flow (Julien, 2006). During slow breathing conditions however, the rhythm of respiration dependent modification decreases the range of LF, such that it closely aligns with the rhythm of respiratory sinus arrhythmia (Russo et al., 2017). The final metric that is sometimes used, which is simply a calculation, is the Baevsky Stress Index (SI) (refer to Figure 2 (Baevsky and Chernikova, 2017)). This is a geometric method to assess IBIs and represent the function of the sympathetic nervous system. Although HRV is often used as a measure of autonomic activity, its use comes with limitations. Variations in heart rate are controlled by both sympathetic and parasympathetic systems, and as a result, selecting biomarkers of solely one system from one’s HRV is difficult. There are, however, some HRV biomarkers that work best in different contexts. SDNN for instance is a reasonable approximation of parasympathetic activity at rest, while RMSSD and LF are better suited to measure parasympathetic activity during slow breathing conditions, such as during biofeedback (Thomas et al., 2019). Even so, there still remains contention if these biomarkers are truly accurate or not, especially due to the aforementioned co-existence of the sympathetic and parasympathetic systems (Shaffer and Ginsberg, 2017).

### 2.4 Data extraction and analysis

Records from each database search were screened for inclusion by two independent authors, with discrepancies being discussed and resolved by mediation by a third author as required. Relevant data from the full-text articles were extracted independently then compared. Due to the heterogenous design as well as the limited number of studies available to be statistically combined, meta analysis was not performed. A narrative scoping review was therefore conducted on the included studies, allowing the reviewers to assess the role of HRV Biofeedback as a potential therapy for FGID and examine the protocols employed by each of the studies per the study aims in order to guide future research in this field.

## 3 Results

### 3.1 Literature search results

**FIGURE 3:**
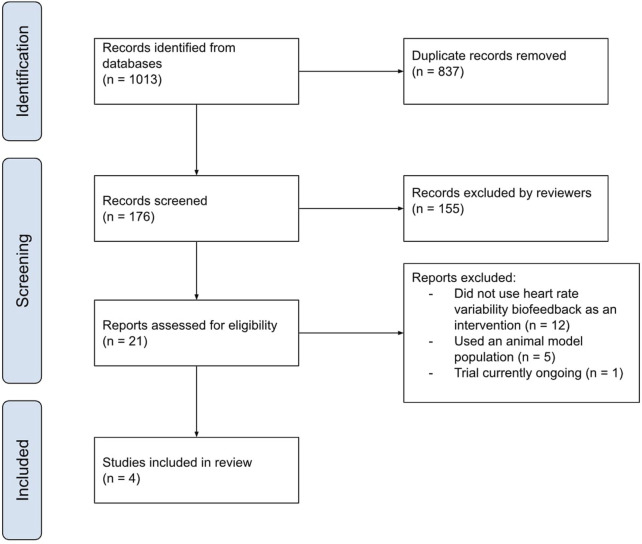
A graphical representation of the screening process of the articles retrieved for this review completed by both independent reviewers.

This literature search had resulted in a total of 1,013 articles (including duplicates) with the following breakdown: 80 results for functional abdominal bloating, 104 results for functional abdominal pain, 90 for functional constipation, 38 for functional diarrhoea, 95 for functional dyspepsia, 71 for gastroparesis, 252 for irritable bowel syndrome and 283 for nausea and vomiting syndrome. The titles and abstracts of these articles were then screened independently by both reviewers according to the exclusion criteria as well as removing duplicates. This resulted in four total articles with some of them assessing multiple of the disorder subtypes that were included in the literature search (2 for functional abdominal pain, one for functional constipation, and three for irritable bowel syndrome). The data from these papers was then extracted and analysed. A graphical summary of the systematic literature review is presented in Figure 3.

### 3.2 Article characteristics

From the four relevant studies identified in the literature search, three were conducted in the USA (Ebert, 2012; Katherine Jurek et al., 2022; Stern et al., 2014) and one in China (Liu et al., 2022). The majority of these addressed IBS, while some studies addressing functional abdominal pain, functional constipation, as well as other subtypes, were found to be lacking (Table 2).

**TABLE 2:** Summary of the key data from the four studies that resulted from the literature search and inclusion and exclusion criteria.

Study	Subtype	Population	Study design	HRVB protocol	Symptom scoring tool	HRVB group results	Control group results
Stern et al. (2014)	Functional abdominal pain and irritable bowel syndrome	24 paediatric participants, including 13 diagnosed with IBS and 11 diagnosed with FAP	Clinical replication series. No information was given on the time frame of the study	Phase 1: Diaphragmatic breathing Phase 2: Resonant Frequency was established, Phase 3: Patients were instructed to breathe at their resonant frequency	Symptom frequency and severity by self report. Full remission was no symptoms for at least 2 weeks. Partial remission was classed as having 50% improvement in symptoms with no impairment in daily functioning for at least 2 full weeks	69.2% of IBS patients achieved full remission and 30.8% partial remission after an average of 8.15 HRVB sessions (range 3–18). For FAP patients, 63.6% achieved full remission and 36.4% partial remission after an average of 10.36 HRVB sessions (range 4–19). There were no age or gender differences, except that females participated in more sessions	Did not use a control group
Ebert (2012)	Functional abdominal pain	26 paediatric participants with diagnosis of FD, IBS, FAP, EGE and functional constipation	Non-randomised interventional trial	Participants found their optimal breathing rate and practised biofeedback at home for 10 min daily using an app. In the remaining sessions, they self-paced for 5 min. Training in the clinic was limited to 8 sessions	Number of days per week with abdominal pain along with pain intensity scales such as Visual Analogue Scales	Pain intensity significantly decreased from a mean score of 5.54 to 2.42 after HRVB, with 54% of participants showing over 50% improvement. SDNN increased significantly from 49.95 to 70.77, and RMSSD from 49.28 to 60.67. No significant changes were found for LF and HF, though they trended as hypothesised. LF/HF ratio increased over the study	Did not use a control group
Liu et al. (2022)	Irritable Bowel Syndrome, functional constipation	85 participants who met the Rome IV criteria for IBS-C	Randomised control trial	Participants had to complete 30 min of SDB for at least 5 days per week while the control group just breathed normally. SDB was done at 6 breaths per minute	The IBS-SSS tool, stool consistency and the weekly frequency of complete spontaneous bowel motions as well as spontaneous bowel motions was recorded. High resolution manometry was also performed	No significant change in IBS-SSS score from baseline, but a significant decrease compared to controls. No significant differences in BSFS, SBM frequency, and CSBM frequency, though SDB showed improvements. Rectal sensitivity and threshold for first sensation were significantly higher in SDB at week 6, but not at week 3. HF, SDNN, and RMSSD increased at weeks 3 and 6. No significant differences in SI.	IBS-SSS mean increased non-significantly. BSFS, CSBM, SBM, and anorectal manometry, HF, SI, SDNN, and RMSSD results showed no significant change, and no significant differences between experimental and control groups
Katherine Jurek et al. (2022)	Irritable bowel syndrome	13 adults with a formal diagnosis of IBS	Randomised control trial	No information on the SDB protocol was given other than the first session was completed in the lab and the rest at home for a minimum of 20 min, at least 4 days a week	IBS-SS	IBS-SSS was unaltered over both groups with no significant differences between groups or within groups. Control was 28 to 22 while Breathing was 24 to 23	IBS-SS showed no significant change in the control group in weeks 2 and 4 compared to the baseline. The same is shown for all HRV metrics recorded

NB: The diagnostic criteria used to classify the FGID, subtypes was only stated in Stern et al. who used ROME III (Drossman, 2006).

IBS-C (Irritable Bowel Syndrome with Constipation).

FAP (Functional Abdominal Pain).

FD (Functional Dyspepsia).

EGE.

SDB (Slow Deep Breathing).

SDNN (Standard Deviation of N-N Intervals).

RMSSD (Root Mean Square of Successive R-R Differences).

LF (Low Frequency Range of Spectral Analysis of Heart Rate Variability).

HF (High Frequency Range of Spectral Analysis of Heart Rate Variability).

BSFS.

SBM (Spontaneous Bowel Motion).

CSBM (Complete Spontaneous Bowel Motion).

SI (Baevsky Stress Index).

Two of the studies were randomised control trials (Katherine Jurek et al., 2022; Liu et al., 2022) while the other two were interventional studies where HRV biofeedback was not compared to a sham control (Ebert, 2012; Stern et al., 2014). These four articles also varied in terms of the target population, with two focused on paediatric populations (Stern, Guiles, and Gevirtz, 2014; Ebert, 2012) and the other two on adult populations (Katherine Jurek et al., 2022; Liu et al., 2022) (Table 2)

All of the included studies used the same primary metrics when quantifying the HRV present, relying on time and frequency domain metrics, to make inferences of vagal and sympathetic tone (Katherine Jurek et al., 2022). All four studies also used ECG as the method of measuring HRV data while participants were in the research laboratory/clinic. For the at-home biofeedback training, Stern et al. used the StressEraser (Helicor Inc, New York, United States of America), a portable, handheld device, which had photoplethysmography (PPG) capabilities. Jurek et al. opted to use a video to guide participants through their biofeedback at home which did not collect heart rate data. Liu et al. and Ebert did not detail any at-home biofeedback practice. All of the four studies used different software to calculate and assess the HRV metrics stated above. Both Stern et al. and Ebert used the J and J Engineering I-330 C-2+ hardware and Stern et al. stated that they used the J and J Engineering USE3 software along with it, which is a combination of hardware and associated software to conduct biofeedback and measure HRV in the lab as well as calculate the metrics related to HRV (Table 3). Jurek et al. instead used a Polar Heart Rate Monitor (Polar Electro Oy, Kempele, Finland) and the raw ECG data from this was then fed through the Kubios software (Kubios Oy, Kuopio Finland), to analyse HRV data. Liu et al. did not state what system they used to analyse HRV data, nor how it was analysed. None of the articles mentioned how they removed any potential artefact from their data as part of their analysis.

**TABLE 3:** Summary of the HRV metrics measured and used as part the respective analysis sections of each of the five studies.

Study	Metrics used	Method of measurement	Method of analysis
Stern et al. (2014)	Heart Rate, Frequency domain spectral analysis	ECG	J and J Engineering I-330 C-2+
Ebert (2012)	SDNN, RMSSD, Frequency domain spectral analysis	ECG	J and J Engineering I-330 C-2+ Portable 6- Channel Physiological Monitoring System
Liu et al. (2022)	Frequency domain spectral analysis, Stress Index, RMSSD and SDNN	ECG	Not stated
Katherine Jurek et al. (2022)	RMSSD, LF/HF, Stress Index, SD2	ECG (Polar Heart Rate Monitor)	Kubios

SDNN (Standard Deviation of N-N Intervals).

RMSSD (Root Mean Square of Successive R-R Differences).

LF (Low Frequency Range of Spectral Analysis of Heart Rate Variability).

HF (High Frequency Range of Spectral Analysis of Heart Rate Variability).

SD2 (A measure derived from a Poincaré plot of heart rate).

ECG (electrocardiogram).

### 3.3 HRV biofeedback protocols

All of these articles used HRV biofeedback as the form of intervention within their exposure groups. There is a considerable amount of variation present in the HRVB protocols being currently used, but the core structure is that it is initially started by providing some participant information to ensure participant buy-in. After this, participants are guided through a biofeedback session of slow, controlled breathing while their heart rate variability is being simultaneously measured. This occurs for a set period of time, while the participant attempts to maintain a mindful state. The remainder of the biofeedback sessions follow a similar format, but may differ if they occur within the clinic or home setting.

The main points of variation across studies emerged when considering the protocols employed for each study. The first point of variation was whether study investigators instruct the participants to breathe at a standardised breathing frequency (i.e. 6 breaths per min) or at the participants’ resonance frequency (Table 4). It is important to acknowledge the distinction between SDB and HRVB used across the studies included in this review. Although they may appear similar, HRVB requires participants to be aware of aspects of their physiology and make conscious efforts to alter it. SDB on the other hand, does not imply this awareness and attempt to control a participant’s physiology. Thus, even if they achieve a similar result, the procedure involved, does have a distinct difference. Another point of variance in the protocol between the four included studies was the length of time during which they conducted the biofeedback training intervention. All four studies appeared to conduct studies of at least 4 weeks or more (excluding Stern et al. and Jurek et al. who did not state the timeframe of their study). The time for which the biofeedback training intervention or SDB intervention was implemented will be categorised by this study as either short-length (70 min weekly or less) or long-length (greater than 70 min weekly). Of the four studies, one falls within the short-length category, while two fall within the long-length category, the one remaining study did not state the weekly duration of training and so could not be categorised. This included time for at-home practice with a pacer device or a smartphone app that paced the individuals breathing along with a measurement of their heart rate via a PPG, averaging 120 min total per week (Table 4).

**TABLE 4:** Summary of the HRV Biofeedback and control protocols used as part the respective analysis sections of each of the five studies.

Study	Total intervention time	Implementation of resonance frequency	Breathing frequency (breaths per min)	Total minimum minutes per week of at-home HRVB training	Control group protocol
Stern et al. (2014)	Not stated	Yes	N/a	140 min	None
Ebert (2012)	Not stated (8 clinic sessions total)	Yes	N/a	70 min	None
Liu et al. (2022)	6 weeks	No	6 bpm	150 min	Regular breathing
Katherine Jurek et al. (2022)	4 weeks	Not stated	Not stated	Not stated	Regular activities

### 3.4 Gastrointestinal outcome measures

The primary outcome measured across all four studies was a change in GI symptoms. The most common symptom scoring tool used was the IBS-SSS (used by Liu et al. and Jurek et al.) (Spiegel et al., 2009), a validated questionnaire that assesses the severity of IBS according to four domains: pain intensity, frequency, location and relation to stool pattern. The remaining studies used symptom frequency and severity as common measures although there was no formal tool used other than a visual analogue scale (Table 5).

**TABLE 5:** Summary of the gastrointestinal symptoms measured and other clinical measures used as part the respective analysis sections of each of the four studies.

Study	Symptom measure	Other clinical measure
Stern et al. (2014)	Self-reported symptom severity and frequency as well as impairment of daily functioning	None
Ebert (2012)	Number of reported days per week with abdominal pain along with pain intensity scales using a visual analogue scale	None
Liu et al. (2022)	IBS-SSS, Stool consistency and weekly frequency of spontaneous bowel motions and complete spontaneous bowel motions	High resolution anorectal manometry
Katherine Jurek et al. (2022)	IBS-SSS	None

Out of the four studies, none used multiple physiological outcome measures. Liu et al. was the only study to include a singular physiological outcome measure of high resolution anorectal manometry.

### 3.5 Study outcomes

Only two of the four studies showed a significant improvement in patient outcomes. Stern et al. and Liu et al. were able to display evidence of the beneficial effects of biofeedback as an emerging therapy. There was a statistically significant decrease in symptom severity and frequency after the biofeedback trial had been completed, compared to baseline; with Stern demonstrating complete remission in 69.2% of participants and Liu et al. showing a statistically significant improvement in IBS-SSS and stool related measures. Both Ebert and Jurek et al. were unable to show symptom improvement, however, these studies were notably heterogeneous in their design and protocol. Due to small sample sizes (n = 24, 26 and 14, with the exception of Liu et al., n = 85), the power of these studies (although not otherwise stated) would be relatively low. In summary, the accumulated evidence from the reviewed papers indicates that biofeedback could be useful as a potential therapy for FGIDs, but more investigation is required to further assess its efficacy.

Jurek et al. stated the compliance of their participants to the SDB intervention, with six out of the seven participants completing at least 80% of their SDB sessions over the 4 weeks with an average of 19 sessions being completed (Katherine Jurek et al., 2022). Stern et al. did not give a measure of compliance to the HRVB intervention but rather stated the number of sessions completed over the study period, which ranged from three to 19 sessions, however they stated that all participants who returned to follow-up experienced some benefit (Stern et al., 2014). Neither Ebert or Liu et al. mentioned compliance to their study intervention. Of the two studies that included a control group (Liu et al. and Jurek et al.) within their protocol, both found no significant changes in the IBS-SSS/IBS-SS sores over the study, compared to baseline.

One of the studies (Liu et al., 2022) found a trend that could be indicative of such a period of time to find efficacy. Within their study, they completed follow ups at weeks three and six after the commencement of the slow, deep breathing exercise (SDB). During these follow-ups, a trend emerged where many of the GI based outcomes measured, only started showing a difference in the SDB group compared to the sham group at the 6 week follow-up and not the 3 week follow-up. This trend was present for the IBS-SSS, BSFS, weekly complete spontaneous bowel motions and weekly spontaneous bowel motions. The same was found for the HRV metrics, keeping in trend with what would be predicted from a sham group (Refer to Table 2). Jurek et al. also followed a similar trend with their study where they did not show a significant improvement in the recorded metrics at their 4 week follow-up mark. These same conclusions cannot be drawn for Stern et al. and Ebert as neither of these studies stated their follow-up periods for their participants.

Of the four studies, three of them provided a summary of the participants’ HRV profiles at various points of their studies. Ebert showed a marked improvement in their participants’ HRV profiles with increases in RMSSD and SDNN showing a statistically significant change at the end of the study, compared to the initiation (p < 0.05). Liu et al. also provided a summary of their participants HRV profiles throughout the study, and found that the interventional group experienced increases in their RMSSD, SDNN and HF compared to the control group, with the change in HF being statistically significant. Jurek et al. found no significant changes in the LF/HF, SNS index or PNS index in the interventional group compared to the control group across the course of the study. It is to be noted that none of the studies stated whether these measurements were taken at a participant’s baseline or during a HRVB/SDB session.

## 4 Discussion

This scoping review has systematically evaluated the current literature regarding HRVB and its potential use as a therapy for FGID, with a particular focus on the protocols and outcomes each study has employed. The studies identified had a heterogeneous design, with half being randomised controlled trials and the other being non-randomised interventional studies. Half of the studies identified, showed that HRVB had a beneficial effect on FGID symptoms, however, significant heterogeneity was identified across all studies. This review highlights the potential role for HRV biofeedback in FGIDs, while highlighting that duration of biofeedback training as a potential key parameter for treatment efficacy and providing guidance for advancing future studies based on the existing literature.

### 4.1 Resonance frequency

One key point of variation emerging from this review is whether study investigators instructed the participants to breathe at a standardised frequency breathing as used in SDB protocols (i.e. 6 breaths per min) (Liu et al. and Juek et al.) or at the participants resonance frequency as used in the HRVB protocols (Stern et al. and Ebert). The research into the benefit of employing resonance frequency into biofeedback training is limited, although an analogue study conducted in 2017 found that using a resonant frequency compared to a standardised breathing frequency was associated with a higher positive mood and a significantly higher LF/HF HRV ratio, a theorised surrogate of parasympathetic/sympathetic activity (Steffen et al., 2017). There is a standardised methodology for finding one’s resonant frequency, which typically involves trialling several different breathing frequencies for a short period of time and assessing the resultant LF power, HR_max_ - HR_min_, and participant comfort to find the optimum frequency (Lehrer et al., 2013; Shaffer and Meehan, 2020). Variations in resonant frequency can be influenced by one’s height and sex, with taller individuals and men having lower resonance frequencies than shorter individuals and women. However, most people tend to have resonant frequencies within a tight range of 5–6.5 breaths/min (Lehrer, 2013; Vaschillo et al., 2006). There is some evidence that one’s resonant frequency is not a stable metric, with one study finding a change in resonant frequency with 66.7% of its participants (Capdevila et al., 2021); however this was limited to a change in the mean of 0.2 breaths/min. When considering that most biofeedback systems are only able to adjust the breathing pacer in 0.5 breaths/min increments, this change in resonant frequency between tests may be clinically negligible. The results of this review do not, unfortunately, gleam the benefit of resonance frequency, with an equal distribution of resonant frequency breathing and standard frequency breathing between studies that showed improvements in gastrointestinal symptoms post biofeedback. However, the sample size of this review is small and previous research suggests that resonant frequency is a viable method to use to optimise biofeedback training. As such it is suggested that more research is required into the topic to further understand the extent of its benefit, particularly where it applies to gastrointestinal disorders.

### 4.2 HRV measurement and analysis

All the studies employed the use of ECG in the clinic/lab setting to monitor the HRV of participants during the biofeedback exercises. ECG (often measured over 24 h) is considered the gold standard of HRV measurement (Shaffer and Ginsberg, 2017). However, a question has emerged about the validity of other forms of HRV measurement. The main contender to the ECG is the PPG method which was used for HRV measurement during at-home biofeedback sessions in Stern et al. This method relies on a light source emitting into the participant’s peripheral artery (i.e., radial artery or digital artery), a proportion of this light is then absorbed according to the volume of blood in the artery at any one time, and the rest is reflected back towards the PPG device to be sensed as used in Stern et al. Because the volume of blood in the artery varies in accordance with the cardiac cycle, the PPG gives a reliable measure of the pulse rate and thus, by extension, the heart rate (Alian and Shelley, 2014). This method is much more portable and accessible than ECG, with its key drawback being that the ECG allows for better theoretical detection of ectopic beats with its ability to show the electrical activity of the heart. However, a recent study shows physicians were able to detect atrial fibrillation using PPG measurement with equivalent accuracy to single-lead ECG (Gruwez et al., 2021), and therefore PPG may be feasible for use during HRVB sessions. PPG also has the added benefit of being conducted with a participant’s smartphone using its inbuilt flash and camera, therefore being highly accessible, and that this method, when combined with applications that employ the biofeedback principles, is able to detect HRV to accurately (Plews et al., 2017; Vandenberk et al., 2017). This altogether, opens up the possibility of conducting HRVB sessions outside of the clinic/lab setting using PPG, increasing patient’s access to HRVB, and allowing further research to explore its use in different settings with comparable reliability. Further research into the use of HRVB using at-home PPG modalities is recommended.

In terms of how the studies chose to analyse their HRV data through their trials, there appears to be a lot of commonality between the studies in terms of how they measure, display and analyse HRV metrics. All of them calculated a spectral analysis to display the power of the different frequency domains; three of them measured SDNN and RMSSD, and two of them calculated some form of stress index, all of which have some relation to autonomic activity (Thomas et al., 2019). The studies also conducted a spectral analysis of an individual’s HRV data via a Fast Fourier Transform, which also provides useful information about one’s autonomic functions, with changes in power in certain frequency bands being related to changes in sympathetic and parasympathetic activity (Shaffer and Ginsberg, 2017). This spectral analysis is often expressed as a ratio of LF/HF to analyse the balance of the sympathetic and parasympathetic systems. The assumption behind this is that LF power and HF power both correspond to sympathetic and parasympathetic activity respectively (Pagani et al., 1984; Shaffer et al., 2014). However, as mentioned earlier, this has been challenged in the past as the SNS and PNS are not solely influenced by LF and HF power. There is often some cross-over between them along with confounding due to baroreflex activity and respiration mechanics (Billman, 2013). This is supported by the evidence that SDNN and RMSSD are commonly used metrics to describe the variation in heart rate with both of them being strongly correlated to autonomic activity and its influence on heart rate and respiratory sinus arrhythmia (Shaffer et al., 2014; Shaffer and Ginsberg, 2017). Both of these are also greatly correlated to the spectral analysis of heart rate, with SDNN being associated with changes in ULF, VLF and LF power and RMSSD being highly correlated to HF power.

The SI (Figure 2) is another measure that is used as an analogue of sympathetic activity. First developed by Baevsky, this metric is highly sensitive to changes in sympathetic tone both within emotional and physical stress situations (Baevsky and Chernikova, 2017). SI has been validated within its use in psychosomatic self-regulation, although evidence of its validation in biofeedback studies is scarce (Ognev et al., 2019).

In addition, although there are many time periods over which HRV is measured and these metrics can be calculated, the standard minimum period of time required to get a measurement of any of these is 5 minutes (Shaffer and Ginsberg, 2017). However, none of the studies specified whether measurements of HRV for participants were collected during the HRVB/SDB sessions or the periods of time outside this. The omission of this detail introduces a degree of confounding when comparing the effect of the interventions used on participant’s HRV, as one’s HRV can vary with the context in which it is being measured.

Overall, the measurement and analysis of HRV during HRVB sessions appears to be well explored and comparable across all studies, which aids interpretation across studies, despite differing HRVB protocols.

### 4.3 Length of heart rate variability biofeedback therapy

Across all studies included in this review, there emerges a trend between the length of HRVB therapy implementation and the improvement in GI symptoms, with improvements occurring at the minimum 6 weeks of therapy. This finding was demonstrated when examining both Jurek et al.‘s and Liu et al.‘s trials. Jurek et al. only had their participants practise biofeedback for 4 weeks while Liu et al. had their participants practise SDB for 6 weeks. Where Jurek et al. did not find any significant improvements in symptoms, Liu et al. did. And upon closer inspection into Liu et al.‘s findings, these differences only started to become significant after 4 weeks into Liu et al.‘s trial. Thus, it is possible that both consistency and duration of biofeedback training is an important factor predictive of improvement of clinical FGID symptoms. However, the evidence behind this theory is drawn from only two trials and thus more evidence is needed to support it. The remaining two studies (Ebert and Stern et al.) do not state how long their follow up periods are and so we are not able to draw this conclusion from them. There was noticeable protocol variance across studies that introduces confounding into this suggested correlation effect. Thus, even though it is possible that there is a 6 weeks minimum therapy period needed to observe improvements in symptoms, it is also possible that with improvements in overall protocol, this might be reduced. However, it holds true that consistency of HRVB practice improves its effectiveness outside of each session, although the extent to which this affects the gastrointestinal system is still yet to be explored (Lehrer et al., 2003; Wheat and Larkin, 2010). Due to this, it is recommended that HRVB therapies be used within a regular format rather than as a single session to improve their efficacy.

It is also to be stated that the two long-length studies (Stern et al. and Liu et al.) found statistically significant improvements with their protocol compared to the single short-length study (Ebert). This does suggest that practicing HRVB for over 70 min in a week could provide more of a benefit than a shorter length of training. This is however based on a small sample size of studies, and more research will be needed to confirm the accuracy of this conclusion.

### 4.4 Symptom measurement

All four studies identified had similarities in having a strong focus on the symptoms associated with the specific FGID subtype they were investigating. Only one of the studies conducted a clinical, specifically anorectal manometry, in order to identify the changes in the threshold of anorectal sensation, providing a more objective assessment of gastrointestinal physiology than symptoms alone (Liu et al., 2022). This focus on symptoms is likely due to the historic focus on the symptoms of FGID, such as in the Rome criteria of diagnosis, and relatively little is conclusively known about the physiology underlying these disorders (Rome IV Criteria, 2020). The most common symptom scoring used across studies was the IBS-SSS (used by Liu et al. and Jurek et al.). This scale is well tested psychometrically and is easy to use with a good reproducibility. However, the main drawback of this tool is that it lacks adequate correlation with other abdominal pain measurement tools (Mujagic et al., 2015). Only one of the studies also recorded the impact of these symptoms on the participant’s quality of life (Stern et al., 2014), an impact of these disorders which can sometimes be overlooked by clinicians (Rocque and Leanza, 2015). Overall, the focus on symptomatic effects of FGID is well observed across all studies, but the lack of co-existing physiological markers recorded is noticeable. Future research in the area would benefit from greater use of objective measure of gastrointestinal physiology as well as autonomic physiology, alongside symptom measures where appropriate, as this would allow for greater reproducibility and comparability across studies. There are several currently used methods that provide a more objective measure of physiological gastrointestinal changes such as Gastric Emptying Studies, Colonic Transit Studies and Electrogastrography; all of which can be used as potential outcome measures for similar future trials. There are also new methods that are being developed, giving researchers and clinicians an insight into the physiology of FGIDs and providing biomarkers for analysis. These techniques include Body Surface Gastric Mapping (BSGM) as well as High Resolution Manometry (HRM), both of which are a methods that measure either gastric electrical activity or pressure with high accuracy and correlate it with symptoms, providing a new understanding of the physiological basis of FGID symptoms (Schamberg et al., 2023; Shimamura et al., 2021). With future research being conducted, more focus can be applied to physiological biomarkers of FGID as outcome measures to better understand the effect of HRVB on the underlying physiology.

Across all four studies, it is clear that there exists substantial heterogeneity in HRVB implementation, which may affect its efficacy in FGID populations. Even with trends observed across the few studies included in this review, of a correlation between improvement in GI symptoms and duration of HRVB therapies, more studies need to be conducted using HRVB within FGID populations to better understand how differences in protocols can affect FGID symptom outcomes to inform optimal protocol design and facilitate standardisation.

### 4.5 Interventional and control group results

Out of the four studies, only two of them employed the use of a control group as a comparison for the biofeedback groups results (Katherine Jurek et al., 2022; Liu et al., 2022), while the other two did not employ a control or similar method (Ebert, 2012; Stern et al., 2014). The studies that did use a control group found no statistically significant improvements in any measurements within the control groups at follow-up compared to baseline for both gastrointestinal related outcomes as well as HRV biomarkers. Out of these two studies, one observed an improvement in gastrointestinal outcomes and HRV biomarkers compared to the control group (Lius et al.), while the other did not show an improvement (Jurek et al.). This difference in outcomes could likely be due to differences in protocol as well as therapy duration as Liu et al. conducted HRVB for 6 weeks, compared to Jurek et al. who conducted it for 4 weeks. However, in cases where HRVB has shown to improve measured outcomes, it does so significantly compared to controls.

Only two of the studies found a significant improvement in symptoms after partaking in biofeedback. This is likely due to the heterogeneity of the protocols exemplified earlier in the review. Only two of the studies employed the use of resonance frequency compared to a standardised breathing frequency, and the duration of the biofeedback intervention varies between each study, along with the time that is spent practising biofeedback while at home. One key finding that was found during this review was that it is likely that biofeedback will need to be consistently practised for at least 6 weeks for its effects to become evident. This finding was demonstrated when examining both Jurek et al.‘s and Liu et al.‘s trials. Jurek et al. only had their participants practise biofeedback for 4 weeks while Liu et al. had their participants practise SDB for 6 weeks. Where Jurek et al. did not find any significant improvements in symptoms, Liu et al. did. And upon closer inspection into Liu et al.‘s findings, these differences only started to become significant after 4 weeks into Liu et al.‘s trial. Thus, it is possible that both consistency and duration of biofeedback training is an important factor predictive of improvement of clinical FGID symptoms. However, the evidence behind this theory is drawn from only two trials and thus more evidence is needed to support it. The remaining two studies (Ebert and Stern et al.) do not state how long their follow up periods are and so we are not able to draw this conclusion from them.

Of the three studies that recorded their participants’ HRV profiles at various points of the study, two of them found a statistically significant change in these profiles, trending towards an improvement in HRV profiles for those that completed the intervention. This indicates that practicing HRVB/SDB regularly is able to alter one’s physiology, improving their parasympathetic/sympathetic tone balance. None of the studies stated whether these HRV measurements were taken during a participants baseline period or during HRVB/SDB sessions. This introduces confounding into the interpretation of these results, as HRVB/SDB in itself improves one’s HRV profile during the session, but without consistent measurements across groups, comparing HRV profiles becomes difficult to do. Future research investigating the effect of HRVB/SDB on longitudinal baseline HRV profiles could be beneficial as it would reinforce HRVB’s believed ability to alter one’s long term physiology.

### 4.6 Autonomic regulation techniques

HRVB/SDB is one specific technique that allows the regulation of the autonomic nervous system, shifting the balance of the system away from a consistent sympathetic prominence and allowing for a more flexible system. It does this by synchronising the activation of the vagus nerve during normal breathing with its cyclical activation during the regulation of peripheral blood pressure (Figure 1). Doing this practice frequently, takes advantage of one’s inherent potential capability for neuroplasticity and increases the adaptability and flexibility of the vagal system outside of when an individual is doing these exercises (Wheat and Larkin, 2010). This is important to the management of FGIDs as it targets one of the possible physiological determinants of FGIDs, vagal withdrawal. By “reawakening” the vagus nerve through techniques such as this, and improving one’s cardiovagal modulatory abilities, it opens up the possibility of addressing one of the key factors leading to the manifestation of FGID. Thus, HRVB/SDB becomes part of the puzzle for helping manage patients with these conditions.

There are other ways of activating this same vagal tone outside of solely breathing techniques. These include methods such as aerobic exercise, yoga, rhythmic muscle contractions and progressive muscle relaxation (Albinet et al., 2010; Shaffer et al., 2022; Tee et al., 2022; Tyagi and Cohen, 2016). Each of these techniques improve one’s HRV by improving their vagal tone. HRV is determined by autonomic activity, largely by the rhythmic pulses of the vagus nerve against sympathetic activity, which can be amplified through practices such as these (Baevsky and Chernikova, 2017; Gitler et al., 2022). Thus, during these practices as well as during HRVB/SDB, HRV acts as a measurement tool of autonomic flexibility. An autonomic system with greater inherent flexibility will be able to exhibit more variability in its heart rate when called upon, such as in these exercises. And thus, a greater HRV depicts greater autonomic flexibility, as well as a greater ability to adapt to changes in the environment (Lee et al., 2015).

### 4.7 Limitations of the review

There are several limitations to this review, including its focus on FGID without considering the potential for other disorders both within and outside the gastrointestinal system, such as within the urinary system (Zivkovic et al., 2017). This review also did not assess the variance in baseline HRV for those diagnosed with FGID compared to healthy controls as other reviews have done similar feats (Ali et al., 2023). The differences between SDB and HRVB described in the studies included in this review also provide another limitation. HRVB has a more in-depth learning process for participants as they receive, understand, and influence the real-time representation of their physiology, which can help facilitate better self-regulation as one learns to alter their physiology (Jafarova et al., 2020). It is possible that this improved self-regulation may relate to symptom improvement for FGIDs compared to SDB which does not promote self-regulation in the same way. Finally, we were not able to conduct a quantitative analysis of the studies identified due to the sample sizes of each study as well as the heterogeneity in participant population, FGID subtype, and methodology. These small sample sizes heterogeneity in protocols makes a robust comparison between studies difficult, and thus these results are tentative and hypothesis generating, reliant upon the publication of more research in the area to confirm or deny the trends identified in this review.

### 4.8 Future research

This scoping review, although small, demonstrates that the field of HRVB is promising yet still in its infancy, and thus, more research into its use within the FGID population is needed, particularly more randomised controlled trials to better assess the effect of biofeedback compared to controls. This review is unique to a similar review conducted by Goldenberg et al. which focused on biofeedback solely in the IBS population (Goldenberg et al., 2019). In comparison to Goldenberg et al., this review had a wider focus of FGID subtypes and a tighter focus on specifically HRVB rather than other forms of biofeedback. This review also had the aim of analysing the protocols used across studies to inform an optimal protocol, while Goldenberg et al. aimed to assess the efficacy and safety of biofeedback interventions in the IBS population.

Future studies should also continue to evaluate the use of PPG as a method to measure HRV compared to a single lead ECG, especially when participants are outside of the clinic or lab. PPG has a high level of accessibility and has an ability to be used alongside a smartphone application for biofeedback exercises. And although the benefit of ectopic beat detection is reduced with PPG compared to ECG, the benefit of accessibility could far outweigh the limitations of artefact removal. This makes it a tool that can potentially improve the way that biofeedback is conducted in clinical trials and opens the door to assess how HRV metrics change with each session completed with an almost similar accuracy compared to ECG. This finding, although not unique to this study, is important to recognise in this review, as it improves the accessibility to at-home HRVB interventions, improving methodological guidance for further research.

The utilisation of more clinical tools can further assess the underlying physiology beyond just the symptoms of FGIDs. This could result in a more objective measurement of how an individual’s gastrointestinal physiology changes during the biofeedback intervention, thus allowing for greater advancements in FGID diagnosis and treatment options. Although the size of this scoping review is small, we believe it will act as a valuable resource, highlighting gaps, and informing methodological improvements for further research. The review will likely act to encourage further research in this field, providing guidance to a growing field.

## Data Availability

The original contributions presented in the study are included in the article/supplementary material, further inquiries can be directed to the corresponding author.
